# Supplementary material for: A human kinase yeast array for the identification of kinases modulating phosphorylation‐dependent protein–protein interactions
Source: Mol Syst Biol. 2022 Feb 28;18(3):e10820. doi: 10.15252/msb.202110820 (PMC8883442; doi:10.15252/msb.202110820)
Supplement: Supplementary file 1 — Expanded View Figures PDF [file MSB-18-e10820-s001.pdf]

# Expanded View Figures

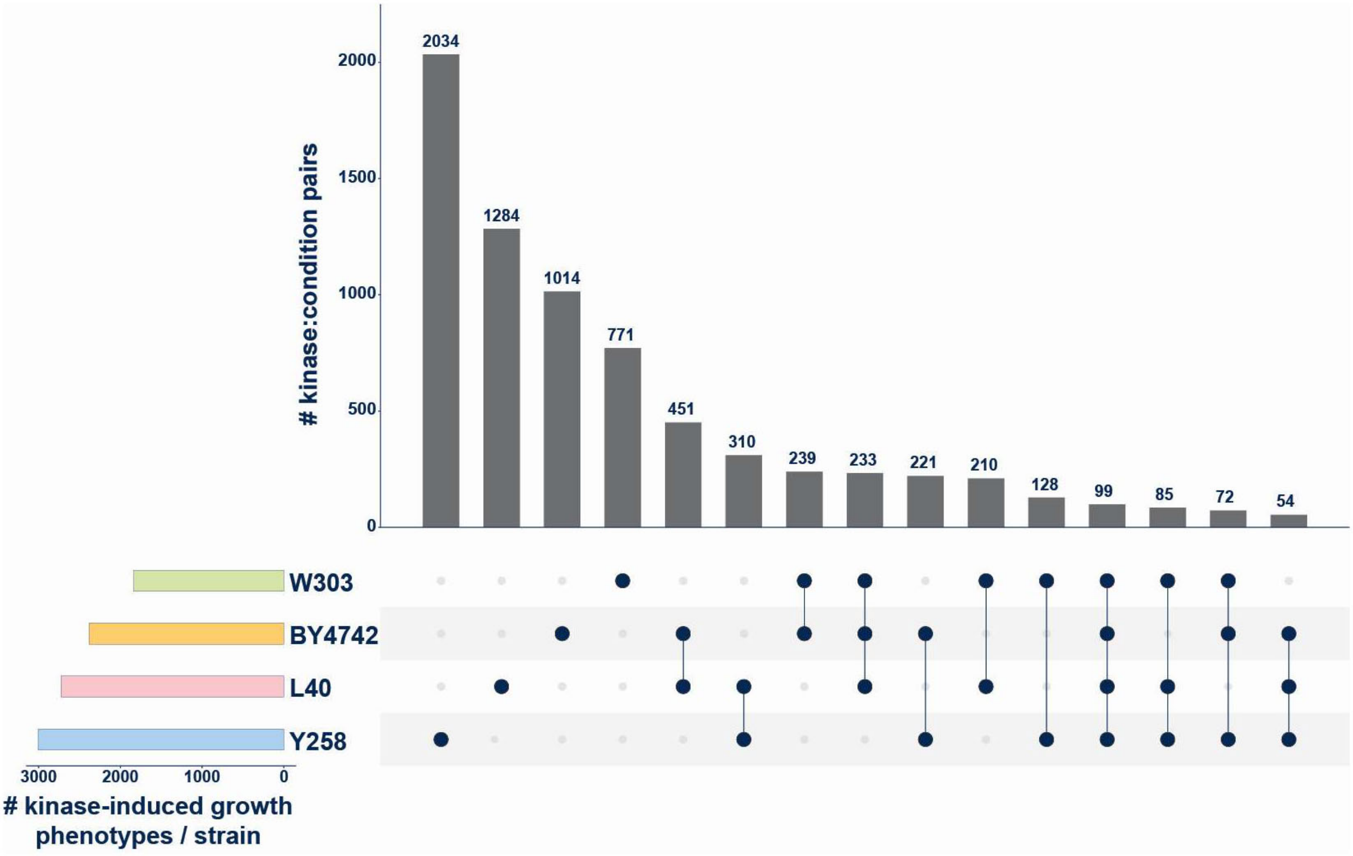


**Figure EV1. Strain sensitivity toward growth effects caused by human kinase expression under different yeast growth conditions.**

Upset plot showing the number of kinase-condition pairs with a growth phenotype across the four strains tested. From the total of 9949 kinase-condition pairs with a growth defect, most were detected in only one strain. Strains Y258 and L40C were more sensitive to kinase-dependent growth perturbation than the diploid W303 stain. 99 kinase-condition pairs with reduced growth were observed in all four yeast strains.


©2022 The Authors

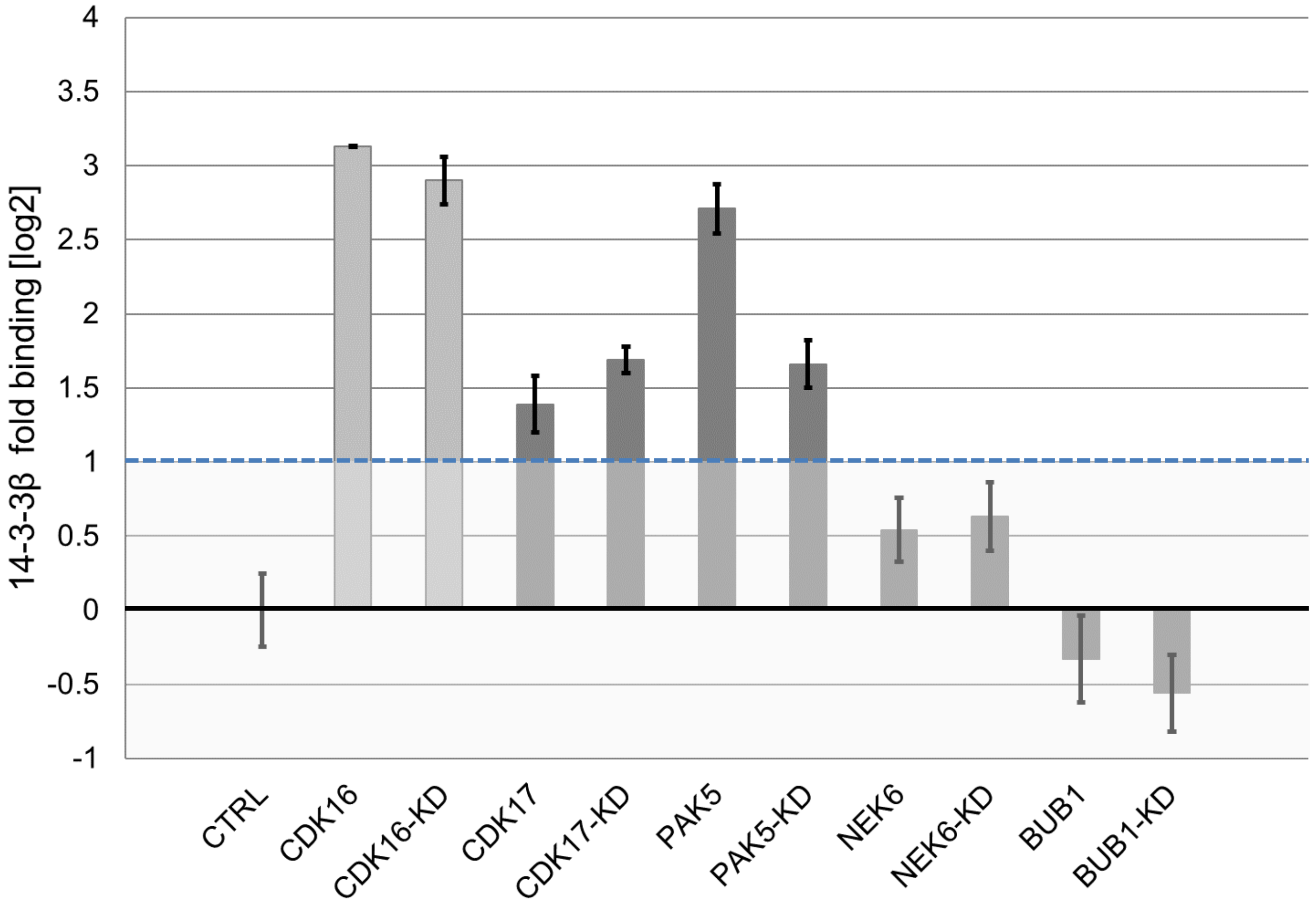


**Figure EV2. Luciferase-based co-immunoprecipitation experiments (related to Fig 6B).**

Results of the LUMIER assay with 14-3-3β (tagged with firefly luciferase) and the indicated kinases (protein A tagged) identified in the Y2H-Screen. CDK16 is a known interaction partner of 14-3-3β and served as positive control (light grey). Error bars indicate SD of triplicate transfections.

**Figure EV3. Transient expression of NEK6 in HEK293 cells does not affect the cell cycle (related to Fig 6E).**

Control experiments with HEK293 cell expressing the indicated NEK6 and NEK6-KD constructs. No significant differences in the proportions of cells in the different phases of the cell cycle can be observed, suggesting that changes in ERα -dependent gene activation cannot be attributed to cell cycle alterations.

A Cells were transfected with the indicated amounts of NEK6 or NEK6-KD plasmids for 24 h and stained with and Hoechst 33258. The cellular DNA content was measured by flow cytometry and the percentage of cells in G1, S, and G2 was calculated using ModFit LT software.

B Representative cell cycle profiles generated by the ModFit LT software for the indicated transfection experiments.

©2022 The Authors

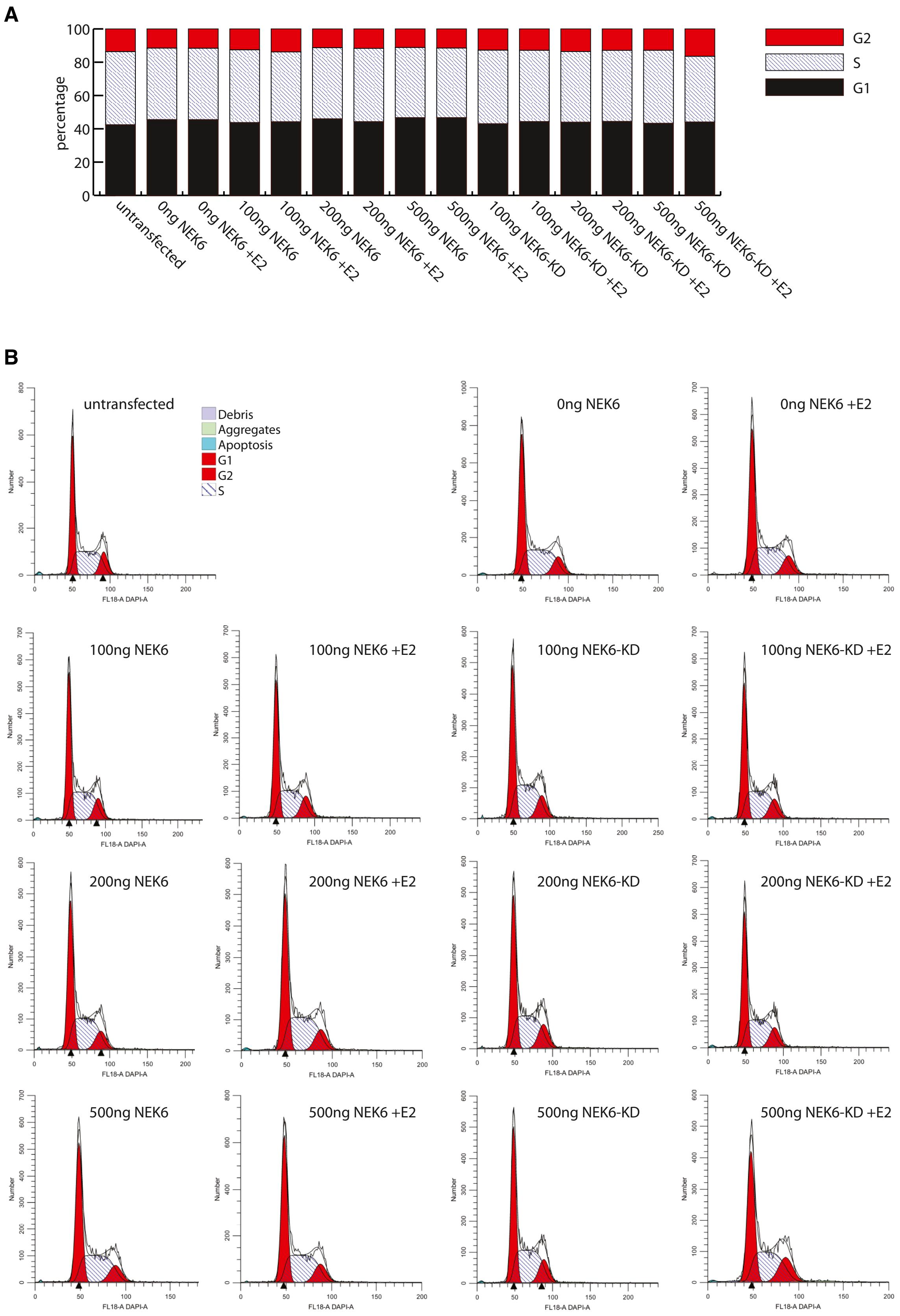


**Figure EV3.**


©2022 The Authors
