# Supplementary material for: A human kinase yeast array for the identification of kinases modulating phosphorylation‐dependent protein–protein interactions
Source: Mol Syst Biol. 2022 Feb 28;18(3):e10820. doi: 10.15252/msb.202110820 (PMC8883442; doi:10.15252/msb.202110820)
Supplement: Supplementary file 6 [file MSB-18-e10820-s003.pdf]

A human kinase yeast array to identify kinases modulating phospho-dependent protein interactions

Stefanie Jehle, Natalia Kunowska, Nouhad Benlasfer, Jonathan Woodsmith, Gert Weber, Markus Wahl, and Ulrich Stelzl
DOI: 10.15252/msb.202110820

Corresponding author(s): Ulrich Stelzl (ulrich.stelzl@uni-graz.at)

Review Timeline:

Submission Date:	19th Nov 21
Editorial Decision:	20th Dec 21
Revision Received:	21st Jan 22
Editorial Decision:	28th Jan 22
Revision Received:	28th Jan 22
Accepted:	31st Jan 22

Editor: Maria Polychronidou

Transaction Report:

(Note: With the exception of the correction of typographical or spelling errors that could be a source of ambiguity, letters and reports are not edited. Depending on transfer agreements, referee reports obtained elsewhere may or may not be included in this compilation. Referee reports are anonymous unless the Referee chooses to sign their reports.)

Thank you again for submitting your work to Molecular Systems Biology. We have now heard back from the three reviewers who agreed to evaluate your study. As you will see below, the reviewers think that the study is interesting and they are overall quite supportive. They raise however a series of (mostly minor) concerns, which we would ask you to address in a revision.

I think that the reviewers' recommendations are clear and therefore there is no need to repeat the points listed below. All issues raised by the reviewers need to be satisfactorily addressed. Please contact me in case you would like to discuss in further detail any of the issues raised.

On a more editorial level, we would ask you to address the following points:

REFEREE REPORTS

Reviewer #1:

Summary
In this study the authors develop a yeast screening assay for kinase activity for a large number of human kinases, including many poorly characterised enzymes. Although working for a large number of kinases, the authors nicely discuss that the screening assay will not work for all kinases due to a variety of reasons, thus acknowledging the limitations of the approach.

The authors also showcase that the assay can be used to identify which kinase(s) that are capable of modulating phospho-regulated interactions. The phospho-yeast two-hybrid assay is a welcome addition to the limited available toolbox for identifying kinases responsible for phospho-switches of protein-protein interactions.

General remarks

I reviewed the manuscript together with a PhD student. We both find that the
study appears well conducted, and the results supports the conclusions. We also find it an important contribution to the field of deciphering the writers of the phosphorylation code, and in linking the actions of kinases to their functional effects on protein-protein interactions.

The study should be of interest to the broad field of cell signalling, and of course in particular for people studying kinases and protein-protein interactions.

Minor points

The yeast strains were cultured in parallel in 384-array format on agar under
various growth conditions. Are there any concerns related to cross-contaminations between conditions? Are there controls for this?
It is stated that the authors removed conditions that reduced growth of more than 175 (17%) colonies. Why is that? It would be helpful if the authors could comment on the rationale for the cut-off, as it is not evident from the text.
What were the control set of proteins without kinase activity used for fig 2? And what was the rational for choosing them, more than not having kinase activity? Having kinase-dead proteins might have been a cleaner design.
What is the sequence specificity for the phospho-Ser/Thr antibodies and how where they chosen?
The authors convincingly showed the application of the phospho-yeast two-hybrid assay. This is a great application, but I miss comments on the scalability of the assay. It would be helpful if the authors can comment on this in the discussion or the conclusions.

Reviewer #2:

In this manuscript, the authors developed an innovative and systematic assay, kinase array, to examine the activities and cellular phenotypes of 266 human kinases under 33 unique treatment (up to 73 conditions) across 4 yeast strains (a total of 243 strain-condition pairs). They were able to identify 150 highly active kinase, including 35 "dark kinases" that are currently quite poorly understood. Furthermore, the authors developed a phospho-Y2H assay to systematically screen for modulation of interactions by kinases (either phosphorylation-dependent interactions or phosphorylation-dependent disruption of interactions). These phospho-Y2H results reveal key cellular functional impact of kinase activities and their substrates; such information are currently quite poorly understood. Overall, their methods and results will have a profound impact to better understand kinase functions, especially for the dark kinases they examined.

The manuscript is well written and easy to follow. The description of the methods and data sources is very clear. All calculations and use of statistics throughout the manuscript were properly carried out.

Major comments

1. For Fig. 1c, the authors maybe can discuss whether using the impact of each kinase in each condition of each yeast strain (in other words, for each kinase in a yeast strain, the color will not be solid any more, but different shades) will improve or reduce the clustering performance. This reviewer is just wondering whether using the raw impacts across all conditions can improve the clustering performance such that we can start to infer which pathways are being affected (maybe not specific substrates) based

on known kinase substrates.

Reviewer #3:

It was a pleasure to read this manuscript. The results were clear, the authors' conclusions were supported by the evidence, and the work overall is of high quality. I have no major issues with this work. I only have a few minor comments that I hope the authors could address in a revised version of the manuscript.

1) The authors could consider adding a figure showing the number of unique phenotypes per condition/strain. That is, were there some conditions (or strains) that were better at picking up growth phenotypes than others? I can imagine that testing more conditions or strains will offer diminishing returns at some point.
2) The data indicate that kinases that are conserved between humans and yeast are more likely to have phenotypes when overexpressed. I found this somewhat surprising. It would be nice to discuss this finding and the likely reasons behind it. For example, do conserved kinases aberrantly phosphorylate the endogenous targets of the yeast orthologs? Or are conserved kinases more likely to be (constitutively) active?
3) Are there any conditions where kinase expression makes the cells grow better?
4) In Figure 2, the authors use DMSO to show that expressing BUB1, NEK6, or activated PKCa slows down yeast growth. In this context, it is unclear why DMSO was used. Was it because DMSO was one of the conditions tested in the original screen and these hits came from there, or was there another rationale? In addition, this experiment is missing a control where no kinase is expressed +/- DMSO to control for the non-specific effect of DMSO on yeast cell growth.
5) I found the analysis in Figure 3 a little cursory. For example, cancer kinases are present in the library but are they overrepresented in the "active" pool of kinases? Similarly, are "dark" kinases or low abundance kinases more or less likely to inhibit yeast growth, compared to other kinases? While it is useful to show overlaps with existing datasets, I think this figure could be moved to the supplements.
6) The authors have also included some "atypical" kinases that are likely derived from the classical Manning et al. kinome review. Further research has shown that most of these proteins are not actual kinases. Rather, the original findings turned out to be false positives (with the notable exception of PI3K/MTOR/ATM family kinases). This might warrant a mention somewhere in the text.
7) Figures 5C and 6B: growth on non-selective medium is not shown.

Other

1) Figure 1C: Topmost active -> Most active
2) Figure 1D: orthomologs -> orthologs
3) Fig 2B legend: NEK6(K821M) should be BUB1(K821M)
4) Figure 3E: "low abundant kinases" -> "low abundance kinases"
5) Figure 5E: it would be good to replace SD3 and SD5 media labels with nonselective and selective medium (for those who are not yeast experts).
6) Page 5, last paragraph: "amino acids sequence context" -> "amino acid sequence context"
7) Page 6, first paragraph: What does different sensitivity mean in the context of tyrosine kinase expression? Does it refer to different activity levels?
8) Page 8, second-to-last paragraph: SKG2 -> SGK2

Point by point reply to reviews, answers in blue

Reviewer #1:

Summary

In this study the authors develop a yeast screening assay for kinase activity for a large number of human kinases, including many poorly characterised enzymes. Although working for a large number of kinases, the authors nicely discuss that the screening assay will not work for all kinases due to a variety of reasons, thus acknowledging the limitations of the approach.

The authors also showcase that the assay can be used to identify which kinase(s) that are capable of modulating phospho-regulated interactions. The phospho-yeast two-hybrid assay is a welcome addition to the limited available toolbox for identifying kinases responsible for phospho-switches of protein-protein interactions.

General remarks

I reviewed the manuscript together with a PhD student. We both find that the study appears well conducted, and the results supports the conclusions. We also find it an important contribution to the field of deciphering the writers of the phosphorylation code, and in linking the actions of kinases to their functional effects on protein-protein interactions.

The study should be of interest to the broad field of cell signalling, and of course in particular for people studying kinases and protein-protein interactions.

We than the reviewer and her/his PhD student for the assessment of the manuscript and their very supportive remarks.

Minor points

The yeast strains were cultured in parallel in 384-array format on agar under various growth conditions. Are there any concerns related to cross-contaminations between conditions? Are there controls for this?

We have set up the 384 array format using a robotics system (custom made gridding robot from Kbiosystems). A series of controls is implemented in the procedure, including the transfer of MTP-plates without growing yeast as well as plates which give specific growth patterns onto agar. Because our stainless-steel pin tool goes through a four-tier cleaning setup [brush1 with 70% ethanol; ultrasonic bath, brush2 with 70% ethanol; and halogen heater@ 400 °C] every time before handling a new plate, we do not observe any cross-contamination during the yeast gridding and growing procedure. However, the cloning procedures and yeast transformation protocols were carried out through classical benchwork in 96 MTP-well format. Here certain errors are possible and cannot be fully excluded. Notably, as a standard precaution, we have sequenced all highly active kinase plasmids from the stock, which was directly used for yeast transformation and confirmed the identity of all the corresponding ORFs through tag-sequencing.

It is stated that the authors removed conditions that reduced growth of more than 175 (17%) colonies. Why is that? It would be helpful if the authors could comment on the rationale for the cut-off, as it is not evident from the text.

Yes, as we explained that conditions which reduced growth of more than 175 (17%) kinases were excluded from further analysis. A total of 243 condition-strain pairs were considered. These condition stain pairs can be found in the Dataset EV1.

The decision on the cutoff is based on the distribution of the number of kinases showing reduced growth per condition strain pair. For each of the four strains, plotting the number of conditions over the number of kinases with growth phenotype gave a bimodal distribution separating conditions with relative fewer, specific kinase signals from conditions that have a general diminishing effect on yeast growth. The distributions provide the rational for the selected cutoff.

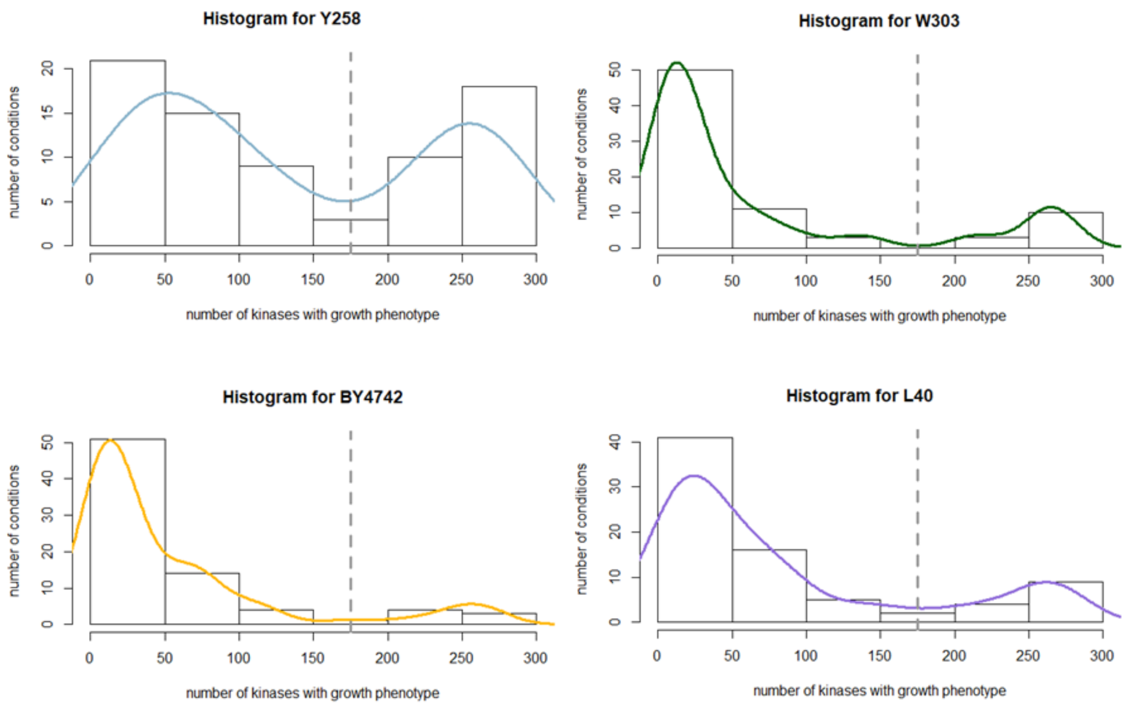

Figure R1:

Histograms showing the number of conditions over the number of kinases causing a growth phenotype under the conditions for the four strains Y258, BY4742, W303 and L40. The colored line represents the distribution of the number of conditions over the kinase count per condition exhibiting a growth phenotype and has a bimodal shape. The grey dashed line indicates the chosen cut-off 175. Conditions with more than 175 kinases that cause a phenotype were viewed as generally growth inhibitory.

We report our reasoning in the main text on page 4 second paragraph in the revised version. "When we plotted the number of kinases showing reduced growth per condition for each of the four strains, we observed bimodal distributions with very low counts between 150 and 200 kinases per condition, separating conditions with relative fewer, specific kinase signals from conditions that have a general diminishing effect on yeast growth. Therefore, conditions with reduced growth of more than 175 (17%) kinases were excluded from further analysis."

What were the control set of proteins without kinase activity used for fig 2? And what was the rational for choosing them, more than not having kinase activity? Having kinase-dead proteins might have been a cleaner design.

We picked some clones from our human ORF library at random, and also included partial kinase ORFs that did not have the intact kinase domain as well as a set of kinases with a mutation rendering them inactive (mainly tyrosine kinases) and empty vector controls. We described in the text and Figure 2A, that this set was useful to determine a background hit rate in the screen and mainly designed following practical reasons. We agree that a good design is to use kinase-dead mutations. This is what we did throughout the manuscript, when assaying kinase activities in non-screening format assays.

What is the sequence specificity for the phospho-Ser/Thr antibodies and how where they chosen?

We chose commercially available standard P-motif antibodies, that show relatively little sequence specificity, as we were detecting the phosphorylation on the yeast proteome. The best example for such an antibody is the 4G10 (mouse monoclonal Ab originally from Upstate), that hardly shows any sequence specificity (Tinti et al. N Biotechnol 2012; doi: 10.1016/j.nbt.2011.12.001) and is very sensitive towards phospho-tyrosine residues in peptides and is therefore widely used in the field of pY research. A similar antibody does not exist for pSerine or pThreonine, that is why we turned to this set of relatively unspecific phospho-Ser/Thr motif antibodies.

We reworded this explanation accordingly in the main text page 6, second paragraph:

"To this end, whole cell lysates from yeast expressing human kinases were subjected to SDS-PAGE and western blotting and probed with either a general phospho-tyrosine recognizing antibody (4G10) or five different, commercially available phospho-substrate antibodies which recognize phosphorylated S/T-sites with little amino acid sequence context specificity (Figure 2C)."

and specified in the Figure legend:

"Commercially available pS/T-antibodies with relatively low sequence specificities were chosen to enable detection of phosphorylation of the yeast proteome by human kinases."

The authors convincingly showed the application of the phospho-yeast two-hybrid assay. This is a great application, but I miss comments on the scalability of the assay. It would be helpful if the authors can comment on this in the discussion or the conclusions.

This is an excellent comment: One of the major advantages of Y2H screen is it scalability, due to easy handling, the use of a high-density matrix format, automatization, and the low false positive rate (biophysical interaction detection). This holds true for our approach and enables the efficient screening of our yeast human kinase array. We have used the phospho-Y2H system in a proteome scale screen to find pY-dependent protein interactions (Grossmann et al. MSB 2015; doi: 10.15252/msb.20145968.). Through pairing pY-binding proteins (SH2 and PTB domain containing) with tyrosine kinases for the query against the human proteome array, this screen was unbiased (Figure R2). The result was to identify phosphorylation-dependent interacting partners, which were implicitly also identified as the kinase phospho-substrate.

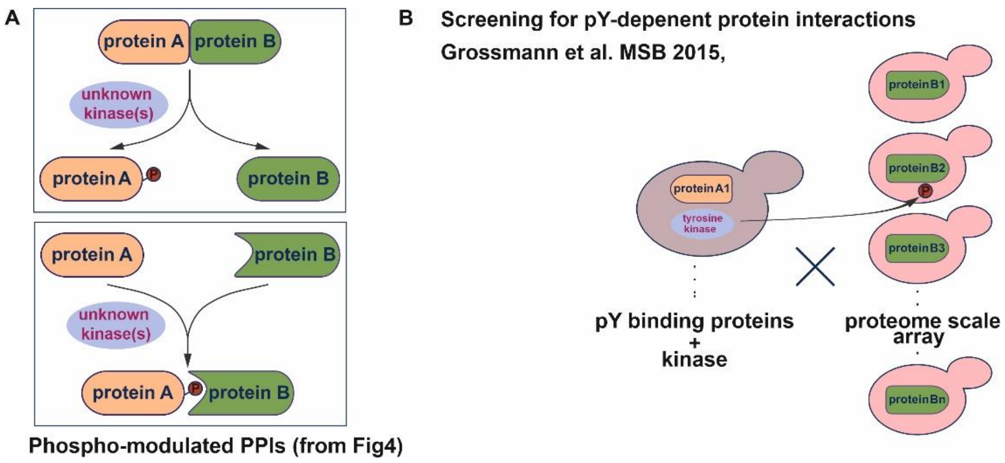

Figure R2

In our approach here, we set out to identify responsible kinases for protein pairs for which it had been known that the interaction was modulated through phosphorylation. This requires substantial prior knowledge which, as we learned in this project, is dispersed in the literature (Figure R3). This requirement is currently the main limitation to scalability. In theory however, the assay is scalable to test any two proteins even without such prior knowledge, to screen for kinases that promote or abolish an interaction. The screening space would clearly be very large in such a setup, and the success rate is expected to be very small.

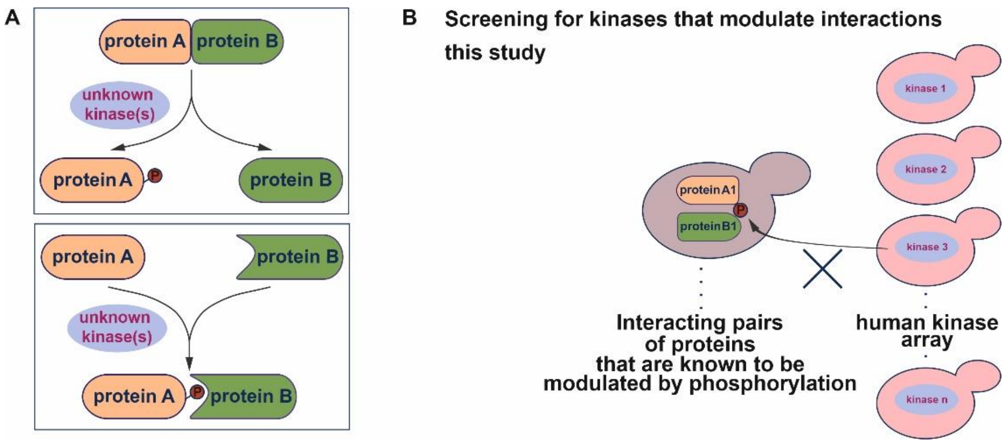

Figure R3

We have amended the text and emphasize that for upscaling of the approach prior knowledge is required. Page 11 last sentence Page12 first.

Reviewer #2:

In this manuscript, the authors developed an innovative and systematic assay, kinase array, to examine the activities and cellular phenotypes of 266 human kinases under 33 unique treatment (up to 73 conditions) across 4 yeast strains (a total of 243 strain-condition pairs). They were able to identify 150 highly active kinase, including 35 "dark kinases" that are currently quite poorly understood. Furthermore, the authors developed a phospho-Y2H assay to systematically screen for modulation of

interactions by kinases (either phosphorylation-dependent interactions or phosphorylation-dependent disruption of interactions). These phospho-Y2H results reveal key cellular functional impact of kinase activities and their substrates; such information are currently quite poorly understood. Overall, their methods and results will have a profound impact to better understand kinase functions, especially for the dark kinases they examined.

The manuscript is well written and easy to follow. The description of the methods and data sources is very clear. All calculations and use of statistics throughout the manuscript were properly carried out.

Major comments

1. For Fig. 1c, the authors maybe can discuss whether using the impact of each kinase in each condition of each yeast strain (in other words, for each kinase in a yeast strain, the color will not be solid any more, but different shades) will improve or reduce the clustering performance. This reviewer is just wondering whether using the raw impacts across all conditions can improve the clustering performance such that we can start to infer which pathways are being affected (maybe not specific substrates) based on known kinase substrates.

In order to address this question, we provide the heatmap from the clustering using the kinase per strain condition counts and added the data to the manuscript as Dataset EV3.

This heatmap reveals six clusters:

Cluster 1 and 2; these two clusters contain very active kinases (57 kinases; median rank 29)

These cluster overlaps well with our group of the most active kinases in Figure 1C.

Cluster 3 represents kinases with moderate activity (51 kinases; median rank 162)

Cluster 4 represents kinases with low activity (51 kinases, median rank 236)

These clusters overlap well with our group of moderately active kinases in Figure 1C.

Cluster 5 and cluster 6 represent the highly active kinases in Figure 1C.

However, in the clustering using the counts (instead of binned counts as in Figure 1C) the grouping is mainly reflecting the kinases activity across the four strains. Cluster 5 is dominated by L40 strain activities (55 kinases; median rank 102), cluster 6 by Y258 strain activities (52 kinases, median 112.5). The clustering results are shown below (Figure R3).

Please note that we added Figure EV1 explaining this result from a different angle. It shows that the L40 and Y258 strains are more sensitive in our screening approach than W303 and BY4742. While the two different cluster approaches agree well, the binned version in Figure 1C favors a more kinase centric view on the activities.

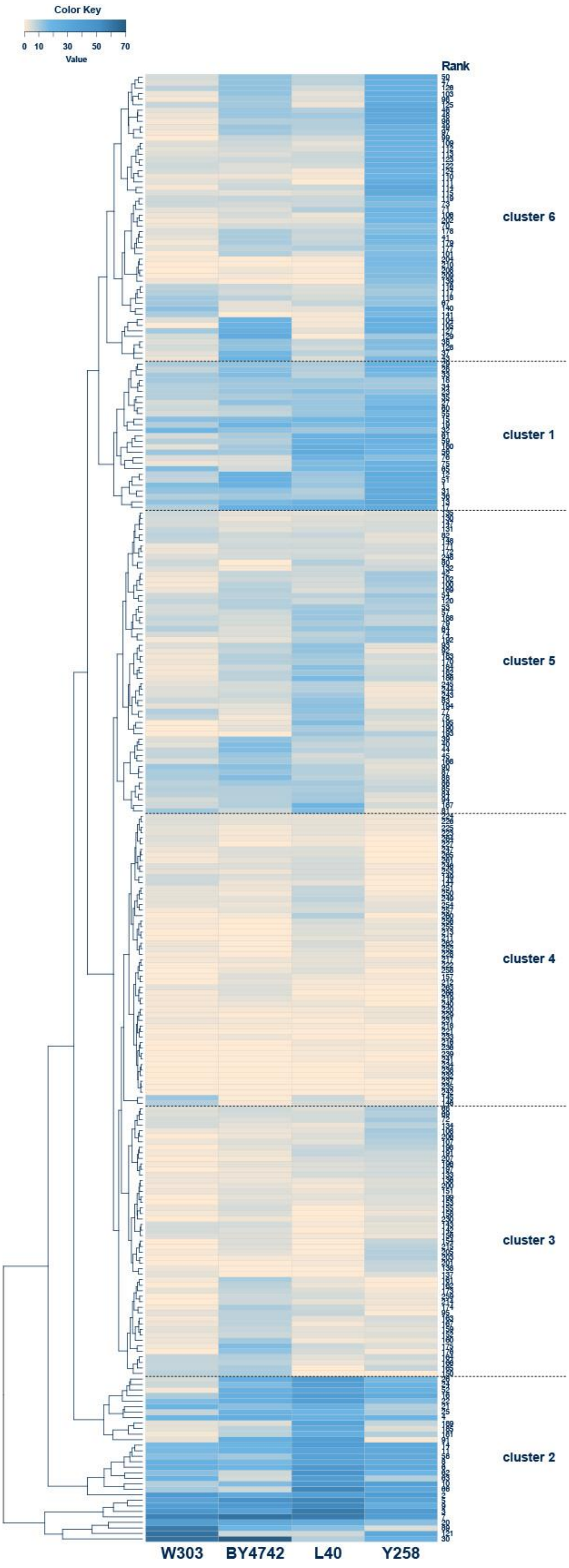
Color Key
0 10 30 50 70
Value
Rank
cluster 6
cluster 1
cluster 5
cluster 4
cluster 3
cluster 2
W303
BY4742
L40
Y258

Figure R4: Hierarchical clustering of the number of conditions with growth phenotype for kinases and strains. Rank (kinases) refers to the clustering results in Figure 1C and is annotated in Dataset EV3.

The suggestion to infer kinase pathway is interesting. As we discussed in the manuscript, there are many factors contributing to whether a human kinase shows activity in yeast or not. To our knowledge there is no yeast phospho-target known that is linked to the growth phenotypes. Putting the human kinases into a pathway context could allow to prioritize for example potential yeast substrates that might be affected. This is a very explorative suggestion and not in the scope of this study.

Reviewer #3:

It was a pleasure to read this manuscript. The results were clear, the authors' conclusions were supported by the evidence, and the work overall is of high quality. I have no major issues with this work. I only have a few minor comments that I hope the authors could address in a revised version of the manuscript.

We thank reviewer 3 for the support of our study.

1) The authors could consider adding a figure showing the number of unique phenotypes per condition/strain. That is, were there some conditions (or strains) that were better at picking up growth phenotypes than others? I can imagine that testing more conditions or strains will offer diminishing returns at some point.

As we are reporting in the text, the use of 4 different strains increased the sensitivity substantially (page 4, end of middle paragraph). Taking the suggestion on board, we now provide an upset plot that directly addresses the reviewer's question. By looking at all 9949 kinase-condition pairs, this figure shows that the L40 and Y258 strains are more sensitive in this approach than W303 and BY4742. It also shows again that most kinase-condition pairs are picked up in one strain only and that the use of multiple strains is important to increase sensitivity.

We present the Figure EV1 showing the number of kinase-condition pairs per strain (main text, page 4, middle paragraph); the data can be found in Dataset EV2.

2) The data indicate that kinases that are conserved between humans and yeast are more likely to have phenotypes when overexpressed. I found this somewhat surprising. It would be nice to discuss this finding and the likely reasons behind it. For example, do conserved kinases aberrantly phosphorylate the endogenous targets of the yeast orthologs? Or are conserved kinases more likely to be (constitutively) active?

Because tyrosine phosphorylation plays only a minor role in yeast, it needs to be separated from S/T phosphorylation for this analysis. We observe that conserved kinases are somewhat more likely to result phenotypes than non-conserved kinases. In line with the comment of the reviewer, we find this result difficult to interpret without further investigation. Both suggestion of reviewer 3 are valuable hypotheses. However, to our knowledge there is no yeast phospho-target known that when phosphorylated by a human kinase is linked to the growth phenotypes. On the other hand, there is anecdotal evidence that human kinases are primed and thus activated by yeast kinases (mentioned in the introduction), but whether there are differences here between conserved and non-conserved kinases and whether it would contribute substantially to our observations remains unclear. A systematic screen to complement yeast gene knock outs with human orthologous protein had a success rate of 47% (Kachroo

et al., Science 2015 10.1126/science.aaa0769), however a similar experiment with non-orthologous proteins has not been carried out yet. Therefore, we can only speculate at this point.

3) Are there any conditions where kinase expression makes the cells grow better?

This is a very interesting question, too. We did not observe any condition where a kinase is promoting yeast growth. However, our screen was not suitable to detect those effects, as we were using fast growing strains and relative long growth times. Answering this question would require a different experimental setup.

4) In Figure 2, the authors use DMSO to show that expressing BUB1, NEK6, or activated PKCa slows down yeast growth. In this context, it is unclear why DMSO was used. Was it because DMSO was one of the conditions tested in the original screen and these hits came from there, or was there another rationale? In addition, this experiment is missing a control where no kinase is expressed +/- DMSO to control for the non-specific effect of DMSO on yeast cell growth.

DMSO is often used as a supplement in chemical genetic screens and we used it in this experiment because it nicely sensitized the yeast strains under liquid growth condition. This can indeed also be seen in the control experiment without kinase. We revised the Figure 2 and added the control experiment with no kinase to the panel B.

5) I found the analysis in Figure 3 a little cursory. For example, cancer kinases are present in the library but are they overrepresented in the "active" pool of kinases? Similarly, are "dark" kinases or low abundance kinases more or less likely to inhibit yeast growth, compared to other kinases? While it is useful to show overlaps with existing datasets, I think this figure could be moved to the supplements.

We present this figure to support the idea that our array can be a versatile tool when combined with various functional readouts. As such we want to show the kinase activities as an overview using a familiar kinome representation. We would like to argue that in this case an overlap analysis is appropriate. To carry out overrepresentation analyses we should probably have a hypothesis why some groups of kinases would be overrepresented. The dark kinase group mainly stems from a research bias. Therefore, it is mechanistically unclear why an unbiased screen in yeast should result overrepresentation of dark kinase. However, good coverage of the dark kinome can be advantageous for drug target research. The argument would be similar for groups of tissue specific and low abundance kinases because expression in yeast is determined by factors largely independent from those in mammalian cells.

6) The authors have also included some "atypical" kinases that are likely derived from the classical Manning et al. kinome review. Further research has shown that most of these proteins are not actual kinases. Rather, the original findings turned out to be false positives (with the notable exception of PI3K/MTOR/ATM family kinases). This might warrant a mention somewhere in the text.

We thank the reviewer for pointing this out. Indeed, we referred to the original kinase definition that was present in the sequence analysis form Manning et. al. We now refer to this issue in the text on page 4 first paragraph and also included a reference: “The set also included five members of the atypical kinases that were shown to differ in some crucial sequence and structural features from the major group kinases (Kanev et al, 2019). “

7) Figures 5C and 6B: growth on non-selective medium is not shown.

Thanks for pointing this out, we have included the agar plates with spots showing growth on non-selective medium in Figure 5C. This information is important as we show that S284E prevents the interaction.

In the experiment shown in Figure 6B, we were searching for a gain of interaction. Therefore, we transferred the yeast directly from YPD to the selective media. As successful mating (diploid formation) is a prerequisite for growth on selective media, we do not routinely transfer the yeast to non-selective

agar in such relatively large experiments. We therefore do not have the corresponding images of plates with the non-selective media. Please note however, that we did several biological replicas and we show three individual mating experiments in Figure 6B.

Other

1) Figure 1C: Topmost active -> Most active

done

2) Figure 1D: orthomologs -> orthologs

done

3) Fig 2B legend: NEK6(K821M) should be BUB1(K821M)

done

4) Figure 3E: "low abundant kinases" -> "low abundance kinases"

done

5) Figure 5E: it would be good to replace SD3 and SD5 media labels with nonselective and selective medium (for those who are not yeast experts).

done

6) Page 5, last paragraph: "amino acids sequence context" -> "amino acid sequence context"

done

7) Page 6, first paragraph: What does different sensitivity mean in the context of tyrosine kinase expression? Does it refer to different activity levels?

Changed to activity

8) Page 8, second-to-last paragraph: SKG2 -> SGK2

done

Thank you for sending us your revised manuscript. We have now evaluated your revised study. We think that the performed revisions have satisfactorily addressed the reviewers' concerns. I am glad to inform you that we can soon accept the study for publication, pending some minor editorial issues listed below.

The authors have made all requested editorial changes.

ACCEPTED **31st Jan 2022**

Thank you again for sending us your revised manuscript. We are now satisfied with the modifications made and I am pleased to inform you that your paper has been accepted for publication.

EMBO PRESS

YOU MUST COMPLETE ALL CELLS WITH A PINK BACKGROUND

PLEASE NOTE THAT THIS CHECKLIST WILL BE PUBLISHED ALONGSIDE YOUR PAPER

Corresponding Author Name: Ulrich Stelzl
Journal Submitted to: Molecular Systems Biology
Manuscript Number: MSB-2021-10820

Reporting Checklist For Life Sciences Articles (Rev. June 2017)

This checklist is used to ensure good reporting standards and to improve the reproducibility of published results. These guidelines are consistent with the Principles and Guidelines for Reporting Preclinical Research issued by the NIH in 2014. Please follow the journal's authorship guidelines in preparing your manuscript.

A- Figures
1. Data
The data shown in figures should satisfy the following conditions:

- the data were obtained and processed according to the field's best practice and are presented to reflect the results of the experiments in an accurate and unbiased manner.
- figure panels include only data points, measurements or observations that can be compared to each other in a scientifically meaningful way.
- graphs include clearly labeled error bars for independent experiments and sample sizes. Unless justified, error bars should not be shown for technical replicates.
- if n< 5, the individual data points from each experiment should be plotted and any statistical test employed should be justified
- Source Data should be included to report the data underlying graphs. Please follow the guidelines set out in the author ship guidelines on Data Presentation.

2. Captions

Each figure caption should contain the following information, for each panel where they are relevant:

- a specification of the experimental system investigated (eg cell line, species name).
- the assay(s) and method(s) used to carry out the reported observations and measurements
- an explicit mention of the biological and chemical entity(ies) that are being measured.
- an explicit mention of the biological and chemical entity(ies) that are altered/varied/perturbed in a controlled manner.

- the exact sample size (n) for each experimental group/condition, given as a number, not a range;
- a description of the sample collection allowing the reader to understand whether the samples represent technical or biological replicates (including how many animals, litters, cultures, etc.).
- a statement of how many times the experiment shown was independently replicated in the laboratory.
- definitions of statistical methods and measures:
  - common tests, such as t-test (please specify whether paired vs. unpaired), simple χ2 tests, Wilcoxon and Mann-Whitney tests, can be unambiguously identified by name only, but more complex techniques should be described in the methods section;
  - are tests one-sided or two-sided?
  - are there adjustments for multiple comparisons?
  - exact statistical test results, e.g., P values = x but not P values < x;
  - definition of 'center values' as median or average;
  - definition of error bars as s.d. or s.e.m.

Any descriptions too long for the figure legend should be included in the methods section and/or with the source data.

In the pink boxes below, please ensure that the answers to the following questions are reported in the manuscript itself. Every question should be answered. If the question is not relevant to your research, please write NA (non applicable). We encourage you to include a specific subsection in the methods section for statistics, reagents, animal models and human subjects.

USEFUL LINKS FOR COMPLETING THIS FORM

http://www.antibodypedia.com
http://1degreebio.org
http://www.equator-network.org/reporting-guidelines/improving-bioscience-research-repor
http://grants.nih.gov/grants/olaw/olaw.htm
http://www.mrc.ac.uk/Ourresearch/Ethicsresearchguidance/Useofanimals/index.htm
http://ClinicalTrials.gov
http://www.consort-statement.org
http://www.consort-statement.org/checklists/view/32-consort/66-title
http://www.equator-network.org/reporting-guidelines/reporting-recommendations-for-tumo
http://datadryad.org
http://figshare.com
http://www.ncbi.nlm.nih.gov/gap
http://www.ebi.ac.uk/ega
http://biomodels.net/
http://biomodels.net/miriam/
http://jjj.biochem.sun.ac.za
https://osp.od.nih.gov/biosafety-biosecurity-and-emerging-biotechnology/
http://www.selectagents.gov/

B- Statistics and general methods

	Please fill out these boxes (Do not worry if you cannot see all your text once you press return)
1.a. How was the sample size chosen to ensure adequate power to detect a pre-specified effect size?	not applicable, reseach did not involve animals
1.b. For animal studies, include a statement about sample size estimate even if no statistical methods were used.	not applicable
2. Describe inclusion/exclusion criteria if samples or animals were excluded from the analysis. Were the criteria pre-established?	not applicable
3. Were any steps taken to minimize the effects of subjective bias when allocating animals/samples to treatment (e.g. randomization procedure)? If yes, please describe.	not applicable
For animal studies, include a statement about randomization even if no randomization was used.	not applicable
4.a. Were any steps taken to minimize the effects of subjective bias during group allocation or/and when assessing results (e.g. blinding of the investigator)? If yes please describe.	not applicable
4.b. For animal studies, include a statement about blinding even if no blinding was done	not applicable
5. For every figure, are statistical tests justified as appropriate?	Statistical test were performed in Figure 1D and 2A.
Do the data meet the assumptions of the tests (e.g., normal distribution)? Describe any methods used to assess it.	yes
Is there an estimate of variation within each group of data?	yes: box plots are shown

Is the variance similar between the groups that are being statistically compared?	yes: box plots are shown

C- Reagents

6. To show that antibodies were profiled for use in the system under study (assay and species), provide a citation, catalog number and/or clone number, supplementary information or reference to an antibody validation profile. e.g., Antibodypedia (see link list at top right), 1DegreeBio (see link list at top right).	commercially available antibodies were used and catalog numbers are provided
7. Identify the source of cell lines and report if they were recently authenticated (e.g., by STR profiling) and tested for mycoplasma contamination.	

* for all hyperlinks, please see the table at the top right of the document

D- Animal Models

8. Report species, strain, gender, age of animals and genetic modification status where applicable. Please detail housing and husbandry conditions and the source of animals.	not applicable
9. For experiments involving live vertebrates, include a statement of compliance with ethical regulations and identify the committee(s) approving the experiments.	not applicable
10. We recommend consulting the ARRIVE guidelines (see link list at top right) (PLoS Biol. 8(6), e1000412, 2010) to ensure that other relevant aspects of animal studies are adequately reported. See author guidelines, under 'Reporting Guidelines'. See also: NIH (see link list at top right) and MRC (see link list at top right) recommendations. Please confirm compliance.	not applicable

E- Human Subjects

11. Identify the committee(s) approving the study protocol.	not applicable
12. Include a statement confirming that informed consent was obtained from all subjects and that the experiments conformed to the principles set out in the WMA Declaration of Helsinki and the Department of Health and Human Services Belmont Report.	not applicable
13. For publication of patient photos, include a statement confirming that consent to publish was obtained.	not applicable
14. Report any restrictions on the availability (and/or on the use) of human data or samples.	not applicable
15. Report the clinical trial registration number (at ClinicalTrials.gov or equivalent), where applicable.	not applicable
16. For phase II and III randomized controlled trials, please refer to the CONSORT flow diagram (see link list at top right) and submit the CONSORT checklist (see link list at top right) with your submission. See author guidelines, under 'Reporting Guidelines'. Please confirm you have submitted this list.	not applicable
17. For tumor marker prognostic studies, we recommend that you follow the REMARK reporting guidelines (see link list at top right). See author guidelines, under 'Reporting Guidelines'. Please confirm you have followed these guidelines.	not applicable

F- Data Accessibility

18: Provide a "Data Availability" section at the end of the Materials & Methods, listing the accession codes for data generated in this study and deposited in a public database (e.g. RNA-Seq data: Gene Expression Omnibus GSE39462, Proteomics data: PRIDE PXD000208 etc.) Please refer to our author guidelines for 'Data Deposition'.<br><br>Data deposition in a public repository is mandatory for:<br>a. Protein, DNA and RNA sequences<br>b. Macromolecular structures<br>c. Crystallographic data for small molecules<br>d. Functional genomics data<br>e. Proteomics and molecular interactions	not applicable
19. Deposition is strongly recommended for any datasets that are central and integral to the study; please consider the journal's data policy. If no structured public repository exists for a given data type, we encourage the provision of datasets in the manuscript as a Supplementary Document (see author guidelines under 'Expanded View' or in unstructured repositories such as Dryad (see link list at top right) or Figshare (see link list at top right).	all data are provided as Data Sets EV1-4
20. Access to human clinical and genomic datasets should be provided with as few restrictions as possible while respecting ethical obligations to the patients and relevant medical and legal issues. If practically possible and compatible with the individual consent agreement used in the study, such data should be deposited in one of the major public access-controlled repositories such as dbGAP (see link list at top right) or EGA (see link list at top right).	not applicable
21. Computational models that are central and integral to a study should be shared without restrictions and provided in a machine-readable form. The relevant accession numbers or links should be provided. When possible, standardized format (SBML, CellML) should be used instead of scripts (e.g. MATLAB). Authors are strongly encouraged to follow the MIRIAM guidelines (see link list at top right) and deposit their model in a public database such as Biomodels (see link list at top right) or JWS Online (see link list at top right). If computer source code is provided with the paper, it should be deposited in a public repository or included in supplementary information.	not applicable

G- Dual use research of concern

22. Could your study fall under dual use research restrictions? Please check biosecurity documents (see link list at top right) and list of select agents and toxins (APHIS/CDC) (see link list at top right). According to our biosecurity guidelines, provide a statement only if it could.	no
